# A novel *PKD1* variant in a patient with very-early-onset ADPKD

**DOI:** 10.1038/s41439-025-00333-z

**Published:** 2025-11-22

**Authors:** Tomomi Kondoh, Takuma Ando, Yuji Matsumoto, Naonori Kumagai, Yu Tanaka, Naoya Morisada, Kandai Nozu, Yohei Ikezumi

**Affiliations:** 1 Department of Pediatrics, Fujita Health University School of Medicine, Toyoake, Japan; 2 Department of Pediatrics, Kobe University Graduate School of Medicine, Kobe, Japan

**Keywords:** Polycystic kidney disease, Gene expression

Autosomal dominant polycystic kidney disease (ADPKD) is a hereditary disorder characterized by the progressive development of multiple cysts in both kidneys, often leading to renal dysfunction and other organ involvement. Typically, the disease progresses slowly, with symptoms and renal failure generally manifesting during adulthood. Here we report a novel frameshift variant, NM_000296.4:c.7563del, resulting in NP_000287.4:p.(Cys2522AlafsTer98), located in exon 19 of the *PKD1* gene, in a patient with very-early-onset ADPKD.

Autosomal dominant polycystic kidney disease (ADPKD) is the most common hereditary cystic kidney disorder and is characterized by the progressive development of multiple renal cysts that can also affect other organs. As patients age, the number and size of the cysts increase, leading to a gradual decline in renal function. By 60 years of age, approximately half of the patients with ADPKD progress to end-stage kidney disease (ESKD). ADPKD follows an autosomal dominant inheritance pattern, indicating that the presence of a single pathogenic allele is sufficient to cause the disease in both males and females. In some cases, the disease may arise from a de novo mutation even when neither parent is affected. The primary causative genes are *PKD1* (located on chromosome 16p13.3, accounting for approximately 78% of the cases) and *PKD2* (on chromosome 4q21; approximately 15%). In recent years, additional genes associated with ADPKD phenotypes in the absence of *PKD1* or *PKD2* mutations such as *GANAB*, *DNAJB11* and *ALG9* have been identified^1^. Although ADPKD typically manifests in adulthood, it has long been recognized that subsets of patients may present with a more severe disease course, occasionally progressing to ESKD during early life. The term very-early-onset ADPKD (VEO-ADPKD) has been proposed for cases diagnosed in utero, characterized by enlarged, hyperechoic kidneys and/or oligohydramnios, or before 18 months of age, with enlarged kidneys and at least one of the following: hypertension, persistent proteinuria or reduced glomerular filtration rate (GFR)^2^. Here, we report the case of a 3-year-old boy with ADPKD.

This case involves a male infant born at 36 weeks and 5 days of gestation via emergency cesarean section due to maternal hypertension, with a birth weight of 2,580 g. Prenatal ultrasonography performed at 33 weeks of gestation revealed bilateral renal cysts. Postnatal ultrasonography confirmed bilateral renal enlargement and multiple cysts. He was the first child of the family. His paternal grandfather was diagnosed with renal cysts at age 40 and underwent kidney transplantation at age 52 due to ESKD. His father had three siblings, one of whom (the patient’s aunt) had been diagnosed with renal cysts and is currently undergoing medical treatment. The remaining siblings, including the father, were reported to be healthy and had not undergone any prior evaluation.

At 4 months of age, his renal function was reduced, with a creatinine-based estimated GFR (eGFR) of 81 ml/min/1.73 m^2^ and a cystatin C-based eGFR of 63 ml/min/1.73 m^2^. Ultrasonography revealed bilateral renal enlargement (renal length of approximately 75 mm) and multiple cysts (about 30 of various sizes in each kidney). At 1 year of age, follow-up imaging revealed bilateral renal enlargement and multiple cysts, along with numerous hyperechoic foci in both kidneys, suggestive of nephrolithiasis (for ultrasonic image of left kidney, see Fig. 1). At age 2, he experienced a febrile seizure. Brain magnetic resonance imaging revealed no cerebral aneurysms. As of age 3, he has not developed hypertension or proteinuria, and his eGFR has remained around 80 ml/min/1.73 m^2^. Although his father, now approximately 40 years old, remains asymptomatic and has shown no signs of renal dysfunction in annual blood tests, we recommend further evaluation because of the family history, and imaging revealed numerous renal cysts (see family pedigree in Fig. 2). Given the early onset and family history of the disease, targeted genetic testing was performed using next-generation sequencing, and informed consent was obtained from the parents following genetic counseling. Following targeted capture of 187 genes (Supplementary Data) using SureSelect (Agilent Technologies), sequencing was performed on the MiSeq platform (Illumina), and variant analysis was conducted. This analysis identified a novel heterozygous variant, NM_000296.4:c.7563del, resulting in NP_000287.4:p.(Cys2522AlafsTer98), located in exon 19 of the *PKD1* gene. This finding was confirmed using Sanger sequencing. This variant was not identified in gnomAD v4.1.0 (https://gnomad.broadinstitute.org/), ToMMo 61KJPN (https://jmorp.megabank.tohoku.ac.jp), ClinVar database (https://www.ncbi.nlm.nih.gov/clinvar/) or Human Gene Mutation Database (http://www.hgmd.cf.ac.uk/ac/index.php) (all databases were accessed on 1 August 2025), suggesting that it is novel and has not been previously reported in major population or clinical variant databases. The same variant was also detected in the patient’s father. According to the American College of Medical Genetics and Genomics guidelines^3^, the variant was classified as pathogenic. This variant results in a frameshift and introduces a premature stop codon, leading to the production of a truncated protein. Given that *PKD1* is a gene in which loss of function is a well-established pathogenic mechanism for ADPKD, this variant fulfills the criteria for PVS1. Moreover, its absence from major population databases satisfies the PM2 criterion, supporting its rarity and further reinforcing its classification as pathogenic.

**Fig. 1:**
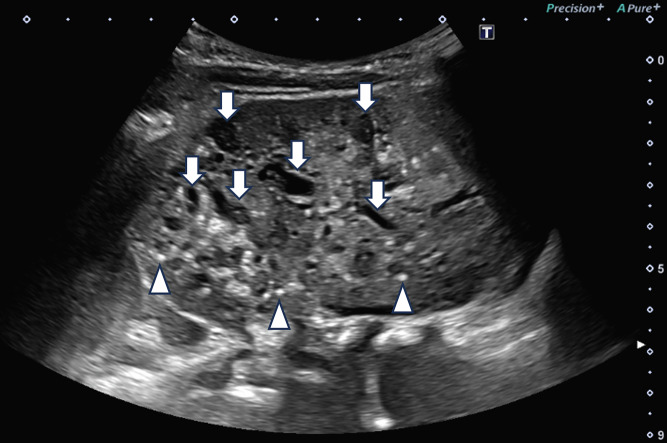
Ultrasound image of left kidney at 1 year of age shows renal enlargement (maximum diameter 96 mm), multiple cysts (with larger ones indicated by arrows) and numerous hyperechoic foci (some marked with arrowheads).

**Fig. 2:**
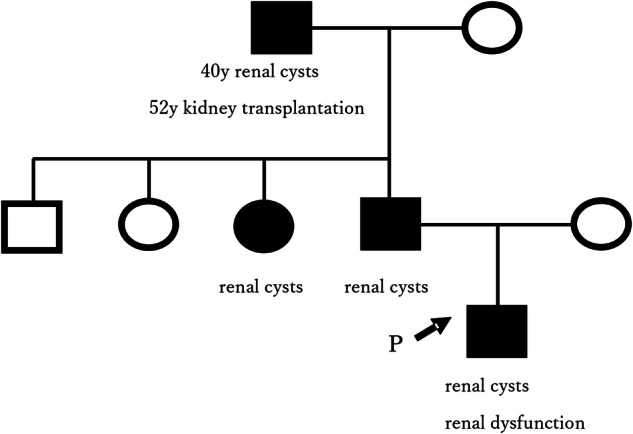
The pedigree of the probands. The proband and his paternal grandfather, father and aunt were diagnosed with ADPKD.

Recent advancements in ultrasonographic diagnostic technology have made the detection of congenital kidney disease during the fetal period increasingly common. Consequently, there is growing interest in the clinical characteristics of VEO-ADPKD, as illustrated by this case, along with intensified research into the genetic mutations associated with early onset.

In adult cases of ADPKD, genetic testing is generally avoided due to well-established imaging-based diagnostic criteria and the complexity involved in sequencing the *PKD1* gene. Based on the international consensus statement developed by the Network for Early Onset Cystic Kidney Disease^4^, simultaneous analysis of a panel of polycystic kidney disease genes is recommended for children with very-early-onset or rapidly progressive disease, regardless of family history. This recommendation reflects the potential for atypical genetic constellations—including biallelic mutations involving hypomorphic *PKD1* or *PKD2* alleles, or co-inheritance with variants in other cystic kidney disease genes such as *TSC2*—that may substantially alter the disease phenotype. So far, aside from atypical genotypic presentations, no specific genotype has been reported to be consistently associated with VEO-ADPKD.

Compared with patients with non-VEO-ADPKD, those with VEO-ADPKD have a significantly higher risk of developing adverse outcomes during childhood, such as renal impairment (eGFR <90 ml/min/1.73 m^2^) or hypertension^5^. Furthermore, 4.3–6.0% of patients with VEO-ADPKD progress to ESKD during childhood, whereas no such progression has been reported in non-VEO-ADPKD cases^5,6^. In the present case, nephrolithiasis developed as early as infancy, despite the fact that kidney stones typically increase in incidence in association with increased kidney volume and renal cyst size, which are generally thought to contribute to urinary stasis^7^. In this case, this finding may indicate a rapid progression of the disease. Aside from atypical genotype–phenotype correlations, no specific genetic variants have been consistently associated with VEO-ADPKD so far. Therefore, the factors contributing to early onset and rapid progression in this case warrant further investigation.

First, regarding the involvement of the novel *PKD1* variant identified in this patient, it is well established that truncating mutations in *PKD1* are associated with more severe phenotypes compared with nontruncating *PKD1* mutations or *PKD2* mutations^1,2^. The frameshift variant found in this case qualifies as PVS1 and may be associated with disease severity. However, the same mutation was also identified in the patient’s father, who did not present with the early-onset form of the disease. Thus, the clinical features observed in this case cannot be explained solely by the *PKD1* truncating variant. It is noteworthy that the paternal grandfather developed ESKD at the relatively young age of 52 and required a kidney transplant, which may suggest a familial predisposition to more severe disease.

Next, modifier genes may contribute to the early onset of renal cysts, particularly in the context of atypical genetic configurations such as biallelic mutations. Notably, one report demonstrated that phenotypic variation between siblings exceeds that observed between monozygotic twins, implicating modifier genes as key contributors to disease heterogeneity^8^. In an analysis of 42 VEO-ADPKD cases targeting *PKD1*, *PKD2*, *HNF1B* and *PKHD1*, 37.2% of patients harbored additional *PKD*-related variants inherited from an asymptomatic parent in addition to the primary pathogenic variant—a significantly higher frequency than the 14.4% observed in adult-onset cases^9^. Another study found that 70% of VEO-ADPKD cases had biallelic *PKD1* or *PKD2* variants using a sequencing panel including 15 cyst-related genes, while the remaining 30% showed only heterozygous *PKD1* variants—suggesting a role for modifier genes^10^. Although a comprehensive analysis of 187 genes in this case did not reveal any definitive candidate genes, the early-onset renal dysfunction observed in the proband, despite the father carrying the same mutation, further supports the involvement of modifier genes. The possibility of yet unidentified modifiers cannot be excluded.

In conclusion, we identified a novel frameshift variant of *PKD1* in a patient with ADPKD who presented with very-early-onset disease. This case underscores the complexity of genotype–phenotype correlations in ADPKD and emphasizes the importance of comprehensive genetic analysis in pediatric patients presenting with atypical symptoms. Further studies are needed to elucidate the genetic and epigenetic factors contributing to disease variability.

## Supplementary information

Supplementary Data A list of 187 cystic kidney disease-related genes included in the comprehensive genetic panel used in the present case.

## Data Availability

The relevant data from this Data Report are available at the Human Genome Variation Database via Figshare at 10.6084/m9.figshare.hgv.3593 (ref. ^11^).

## References

[1] Ali, H. et al. PKD1 truncating mutations accelerate eGFR decline in autosomal dominant polycystic kidney disease patients. *Am. J. Nephrol.* **55**, 380–388 (2024). 38194940 10.1159/000536165 PMC11151966

[2] Cornec-Le Gall, E., Torres, V. E. & Harris, P. C. Genetic complexity of autosomal dominant polycystic kidney and liver diseases. *J. Am. Soc. Nephrol.* **29**, 13–23 (2018). 29038287 10.1681/ASN.2017050483 PMC5748917

[3] Richards, S. et al. Standards and guidelines for the interpretation of sequence variants: a joint consensus recommendation of the American College of Medical Genetics and Genomics and the Association for Molecular Pathology. *Genet. Med.* **17**, 405–424 (2015). 25741868 10.1038/gim.2015.30 PMC4544753

[4] Gimpel, C. et al. International consensus statement on the diagnosis and management of autosomal dominant polycystic kidney disease in children and young people. *Nat. Rev. Nephrol.* **15**, 713–726 (2019). 31118499 10.1038/s41581-019-0155-2 PMC7136168

[5] Nowak, K. L., Cadnapaphornchai, M. A., Chonchol, M. B., Schrier, R. W. & Gitomer, B. Long-term outcomes in patients with very-early onset autosomal dominant polycystic kidney disease. *Am. J. Nephrol.* **44**, 171–178 (2016). 27548646 10.1159/000448695 PMC5098215

[6] Shamshirsaz, A. A. et al. Autosomal-dominant polycystic kidney disease in infancy and childhood: progression and outcome. *Kidney Int.* **68**, 2218–2224 (2005). 16221221 10.1111/j.1523-1755.2005.00678.x

[7] Grampsas, S. A. et al. Anatomic and metabolic risk factors for nephrolithiasis in patients with autosomal dominant polycystic kidney disease. *Am. J. Kidney Dis.* **36**, 53–57 (2000). 10873872 10.1053/ajkd.2000.8266

[8] Persu, A. et al. Comparison between siblings and twins supports a role for modifier genes in ADPKD. *Kidney Int.* **66**, 2132–2136 (2004). 15569302 10.1111/j.1523-1755.2004.66003.x

[9] Audrézet, M. P. et al. Comprehensive *PKD1* and *PKD2* mutation analysis in prenatal autosomal dominant polycystic kidney disease. *J. Am. Soc. Nephrol.* **27**, 722–729 (2016). 26139440 10.1681/ASN.2014101051 PMC4769188

[10] Durkie, M., Chong, J., Valluru, M. K., Harris, P. C. & Ong, A. C. M. Biallelic inheritance of hypomorphic *PKD1* variants is highly prevalent in very early onset polycystic kidney disease. *Genet. Med.* **23**, 689–697 (2021). 33168999 10.1038/s41436-020-01026-4 PMC9782736

[11] Kondoh, T. et al. Data. *Figshare*10.6084/m9.figshare.hgv.3593 (2025).

