# Supplementary material for: A novel PKD1 variant in a patient with very-early-onset ADPKD
Source: Hum Genome Var. 2025 Nov 22;12:28. doi: 10.1038/s41439-025-00333-z (PMC12660832; doi:10.1038/s41439-025-00333-z)
Supplement: Supplementary file 1 — Supplementary Data A list of 187 cystic kidney disease-related genes included in the comprehensive genetic panel used in the present case. [file 41439_2025_333_MOESM1_ESM.docx]

| ACE | BBIP1 | CEP41 | DYNC2H1 | GATA3 | IFT57 | LMOD1 | NPHP4 | SARS2 | TMEM216 | WDR34 |
| --- | --- | --- | --- | --- | --- | --- | --- | --- | --- | --- |
| ACTG2 | BBS1 | CEP83 | DYNC2LI1 | GDF11 | IFT74 | LMX1B | NRIP1 | SCLT1 | TMEM218 | WDR35 |
| AGT | BBS2 | CEP104 | DZIP1L | GDNF | IFT80 | LRIG2 | OCRL | SDCCAG8 | TMEM231 | WDR60 |
| AGTR1 | BBS4 | CEP120 | EP300 | GFRA1 | IFT81 | LRP5 | OFD1 | SEC61A1 | TMEM237 | WNT4 |
| AGTR2 | BBS5 | CEP164 | EVC | GLIS2 | IFT122 | LZTFL1 | PAX2 | SIX1 | TNXB | WT1 |
| AHI1 | BBS7 | CEP290 | EVC2 | GPC3 | IFT140 | MAFB | PAX8 | SlX2 | TOGARAM1 | XPNPEP3 |
| ALG5 | BBS9 | CHD4 | EXOC4 | GREB1L | IFT172 | MAPKBP1 | PBX1 | SlX5 | TRAF3IP1 | ZNF423 |
| ALG8 | BBS10 | CHD7 | EXOC8 | GRIP1 | INPP5E | MKKS | PDE6D | SOX17 | TRIM32 |  |
| ALG9 | BBS12 | CHRM3 | EYA1 | HNF1B | INTU | MKS1 | PIBF1 | SUFU | TSC1 |  |
| ALMS1 | BICC1 | CHRNA3 | FAM149B1 | HOXA11 | INVS | MUC1 | PKD1 | TBX1 | TSC2 |  |
| ANKS6 | C2CD3 | CLCN5 | FAN1 | HOXA13 | IQCB1 | MYH11 | PKD2 | TBX18 | TTC8 |  |
| ANOS1 | C5orf42 | CRB2 | FGF20 | HPRT1 | ITGA8 | MYL9 | PKHD1 | TCTEX1D2 | TTC21B |  |
| ARL3 | C8orf37 | CSPP1 | FGFR1 | HPSE2 | JAG1 | MYLK | REN | TCTN1 | TXNDC15 |  |
| ARL6 | CBWD1 | DACT1 | FGFR2 | HYLS1 | KIAA0556 | NEK1 | RET | TCTN2 | UMOD |  |
| ARL13B | CC2D2A | DCDC2 | FRAS1 | INF2 | KIAA0586 | NEK8 | ROBO1 | TCTN3 | UPK3A |  |
| ARMC9 | CCDC28B | DDX59 | FREM1 | IFT27 | KIAA0753 | NOTCH2 | ROBO2 | TMEM67 | VANGL2 |  |
| B9D1 | CDKN1C | DNAJB11 | FREM2 | IFT43 | KlF7 | NPHP1 | RPGRIP1L | TMEM107 | WDPCP |  |
| B9D2 | CENPF | DSTYK | GANAB | IFT52 | KIF14 | NPHP3 | SALL1 | TMEM138 | WDR19 |  |
